# Bacterial diversity in surface sediments of collapsed lakes in Huaibei, China

**DOI:** 10.1038/s41598-022-20148-0

**Published:** 2022-09-22

**Authors:** Zijian Shen, Zijian Shang, Faxin Wang, Yanhong Liang, Youcun Zou, Fei Liu

**Affiliations:** School of Life Sciences, Huaibei Normal University, Huaibei, 235000 China

**Keywords:** Ecology, Microbiology, Ecology, Environmental sciences

## Abstract

The collapse lake area due to coal mining in Huaibei shows high biodiversity, but the bacterial community composition and diversity in the lake sediments are still rarely studied. Therefore, based on 16S rRNA high-throughput sequencing and combined with analysis of environmental factors, we comparatively analyzed the bacterial community composition and diversity of surface sediments from East Lake (DH) and South Lake (NH) and Middle Lake (ZH) in the collapse lake area of Huaibei. The bacterial community compositions are significantly different in the sediments among Huaibei collapsed lakes, with DH having the largest number of species, and NH having a higher species diversity. Pseudomonadota is the most abundant phylum in the sediments of DH and NH, while the most abundant phyla in ZH are Bacteroidales, Chloroflexales, Acidobacteriales, and Firmicutes. Anaerolineae (24.05% ± 0.20%) is the most abundant class in the DH sediments, and Gammaproteobacteria (25.94% ± 0.40%) dominates the NH sediments, Bacteroidia (32.12% ± 1.32%) and Clostridia (21.98% ± 0.90%) contribute more than 50% to the bacteria in the sediments of ZH. Redundancy analysis (RDA) shows that pH, TN, and TP are the main environmental factors affecting the bacterial community composition in the sediments of the collapsed lake area. The results reveal the bacterial community composition and biodiversity in the sediments of the Huaibei coal mining collapsed lakes, and provide new insights for the subsequent ecological conservation and restoration of the coal mining collapsed lakes.

## Introduction

The collapse lake area of Huaibei due to coal mining is located in the north of Anhui Province. Due to the massive mining of coal resources, massive land collapsed, and many small collapse lakes formed shallow groundwater under the replenishment of rainfall and surface runoff^1^. At present, the area of mined-out subsidence in Huaibei City has exceeded 160 km^2^, while the largest area of lakes formed by stagnant water in the subsidence area is about 36 km^2^. The average depth of these lakes is 2–3 m (the maximum depth is more than 10 m). The difference from natural lakes is that there are many coal gangues at the bottom of the collapsed lake, resulting in complex chemical composition of the sediments, higher heavy metal contents than in normal water environment, and unique aquatic ecosystem^2^. Therefore, the environmental quality of the water and the sediments of coal mining collapsed lakes haves received extensive attention. Investigations show that the research on coal mining subsidence lakes mostly focuses on water quality evaluation, water body and ecological restoration methods, and benthic community structure^3^. However, so far, there are relatively few studies on the bacterial community composition and diversity in the sediments of the Huaibei coal mining subsidence lake area. Thus, the purpose of this study is to analyze the bacterial community composition and diversity in the sediments of the Huaibei coal mining subsidence lake area and to better understand the ecology of this unique aquatic ecosystem.

Understanding and interpreting the relationship between microbial community composition and structures in nature is still an important topic of the scientific field^4^. Microbes are a major component of the ecosystem, and play an important role in maintaining the stability of the environmental ecosystem in areas such as rivers, lakes, estuaries, and oceans^5^. Microbes are also the main driving force of the biogeochemical cycle^6^. According to related studies, the microbial biomass in surface sediments accounts for about 58–88% of the world's total change^7^. Lake sediments provide places for microbial material circulation and energy flow, and changes in environmental factors in sediments and the input of nutrients will affect the composition and structure of bacterial communities^8,9^, including total nitrogen(TN)^10^, total phosphorus(TP), total organic carbon (TOC)^11^, redox potential (Eh)^12^, and pH^13^. At the same time, the contents of heavy metals in the sediments will also affect the activity of bacterial communities. As reports, the environmental differences of sediments lead to appearance of special community characteristics of microbes, such as freshwater or seawater environment, and river or lake environment^14,15^. Hence, it is very important to analyze the community structure and diversity of bacteria in sediments and their relationship with environmental factors, and specific bacterial groups can reflect the status quo of the environment.

At present, high-throughput sequencing has been widely used to analyze the diversity of microbes in various ecological environments^16^. Sadaiappan et al.^17^ found the high-salt sediments in Mad Boon mangrove ecosystems in the Bengal bay were dominated by Pseudomonadota, followed by Acidobacteriales, Firmicutes and Chloroflexales, and uncovered Deltaproteobacteria and Gammaproteobacteria. Kim^18^ found the estuary sediments of Nadong River were dominated by Pseudomonadota, Bacillota, Bacteroidales and Planctomycetes. Deng et al.^19^ studied 6 alkali lakes in Qinghai-Tibetan Plateau based on 454-pyosequecing and found the lake sediments of Tibet were dominated by *methan-otrophs*.

In this study, three representative collapse lakes in Huaibei were selected for collection of surface sediments, including DN, NH, and ZH. These three collapse pit lakes are artificial lakes formed by the collapse of coal mining and the construction of wetland parks around the lakes. Based on Illumina HiSeq 16S rRNA gene high-throughput sequencing, the bacterial diversity, abundance, community composition and bacterial communities of the three lakes were comparatively evaluated, and the physicochemical factors that affected microbial community distributions were identified. This study is aimed to better understand the physiological ecology of coal mine collapse lakes, and to provide some scientific reference for future research on ecological conservation, recovery and utilization of collapse lakes.

## Results

### Physicochemical factors of sediments

The physicochemical indices of 9 surface-sediment samples collected from the three collapse lakes in Huaibei are listed in Table 1. Generally, the surface sediments of collapse lakes are weakly alkali, and pHs of sediments from ZH and NH are significantly different (*p* = 0.004). Neither TN nor TP is significantly different among the collapse lakes (*p* > 0.05). TN concentration is the highest in ZH 1 and lowest in NH 3, which are 0.901 and 0.477 g/kg respectively. TP concentration is the highest in NH 2 and lowest in ZH 2, which are 0.196 and 0.034 g/kg respectively. The As concentrations in surface sediments of DH, ZH and NH are 2.295–4.419, 1.064–2.454, and 1.375–2.433 mg/kg respectively, and are the highest in DH 1 and lowest in ZH 1. The Sb concentrations in surface sediments of DH, ZH and NH are 1.994–2.896, 2.341–6.962, and 1.337- 4.100 mg/kg respectively, and are the highest in ZH 3 and lowest in NH 1. Hence, neither As nor Sb concentrations are significantly different among the surface sediments from different collapse lakes (*p* > 0.05).

**Table 1:** Physical and chemical indexes of sediments in Huaibei collapsed lakes.

Sampling site	pH	TN (g/Kg)	TP (g/kg)	As (mg/kg)	Sb (mg/kg)
DH 1	8.02	0.611	0.129	4.419	2.576
DH 2	8.06	0.665	0.114	2.359	1.994
DH 3	8.10	0.503	0.127	2.295	2.896
ZH 1	8.13	0.901	0.165	1.064	2.746
ZH 2	8.12	0.551	0.034	1.826	2.341
ZH 3	8.26	0.529	0.111	2.454	6.962
NH 1	7.87	0.565	0.103	2.433	1.337
NH 2	8.00	0.522	0.196	1.901	4.100
NH 3	7.95	0.477	0.071	1.375	1.630

### OTU clustering and microbial α diversity of sediments

The microbial diversity of 9 sediment samples collected from the three mining collapse lakes in Huaibei was analyzed (Table 2). Quality control filtration of raw sequencing data yielded 604420 valid sequences, and at the similarity level of 97%, 2034 OTUs were identified, which belong to 34 phyla, 91 classes, 206 orders, 338 families, 584 genera and 603 species. The coverage rates of sample library are all > 99%, suggesting the sequencing results are reliable and can reflect the actual situations of microbial communities in the sediments of the sampling areas.

**Table 2:** Microorganism OTU, sequencing coverage and diversity index of sediment samples.

Sample ID	Effective tags	OTU	ACE	Chao1	Simpson	Shannon	Coverage
DH1	68366	1619	1707.8104	1716.0608	0.9924	8.7039	0.9966
DH2	67063	1597	1674.4301	1671.2182	0.9922	8.6728	0.9967
DH3	67438	1611	1723.1380	1762.1278	0.9923	8.6873	0.9959
ZH1	66402	1087	1126.2087	1168.0698	0.9919	8.3841	0.9982
ZH2	66546	1079	1099.2795	1134.3125	0.9924	8.4277	0.9988
ZH3	64740	1041	1054.0353	1063.0769	0.9915	8.3229	0.9991
NH1	67217	1517	1649.9786	1712.5093	0.9931	8.9891	0.9948
NH2	68296	1515	1667.9128	1756.1212	0.9931	8.9865	0.9947
NH3	68352	1508	1623.2977	1651.375	0.9928	8.9732	0.9954

The number of OTUs in the sediments of DH, ZH and NH are 1597–1619, 1041–1087, and 1508–1517 respectively, with the highest levels from sediments of DH, the ACE and Chao 1 both rank as DH > NH > ZH (Table 2), which is consistent with the order of OTU abundance. The Simpson index and Shannon index rank as NH > DH > ZH, suggesting sediments from NH show higher species diversity.

T-test uncovers the differences in α diversity index among the collapse lakes, and both ACE index and Shannon index are significantly different among the three lakes (*p* < 0.05). The values of Chao 1 index in DH and NH are not significantly different (*p* > 0.05), but are both very significantly higher than those of ZH (*p* < 0.05). The values of Simpson index are not significantly different between DH and ZH (*p* = 0.2411), but are very significantly between DH and NH (*p* = 0.0037) and between NH and ZH (*p* < 0.05).

**Figure 1:**
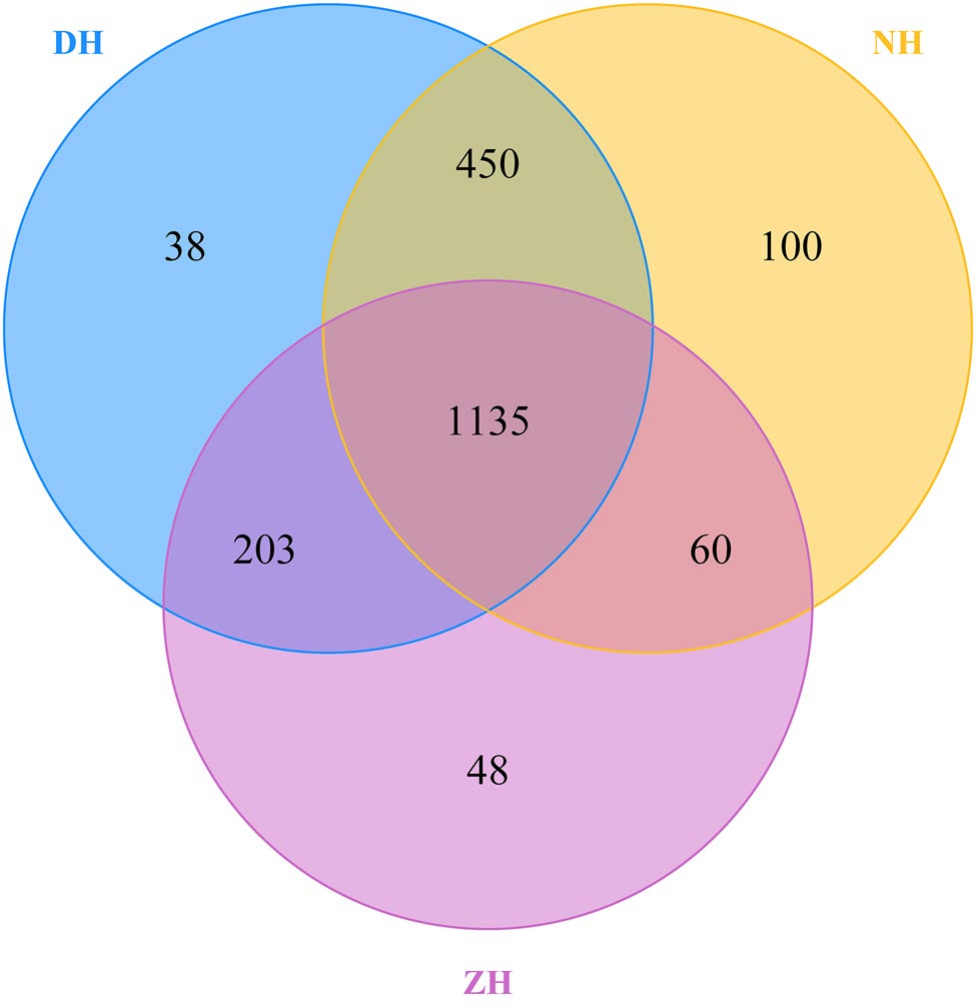
Venn diagram denoting the distribution of OUT.

Venn images show the common OTUs and exclusive OTUs among DH, NH and ZH in Huaibei, and clearly display the between-sample superposition of OTUs (Fig. 1). Totally 2034 OTUs were identified from the 3 collapse lakes, including 1135 common OTUs, which account for more than half of all OTUs. This result indicates the microbial community compositions in sediments of the three collapse lakes are similar. There are 1585, 1338 and 1195 common OTUs between DH and NH, between DH and ZH, and between NH and ZH respectively. DH, NH and ZH have 38, 48 and 100 exclusive OTUs respectively.

### PCoA and UPGMA

**Figure 2:**
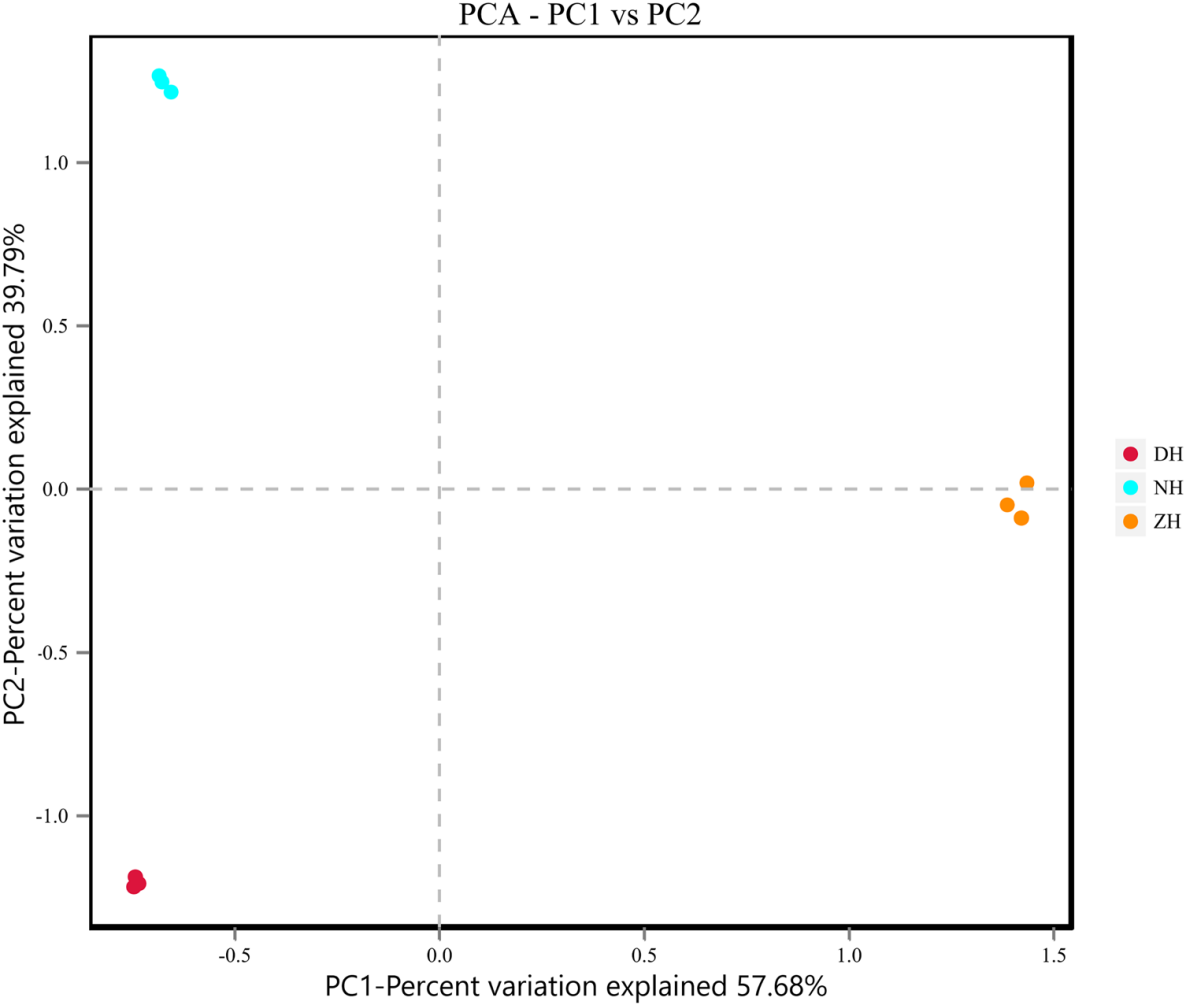
PCA analysis chart.

Based on the four distance matrices obtained from Beta diversity analysis, the PCA results plotted separately using R language are shown in Fig. 2. The two axes can explain 57.68% and 39.79% of the classification results, respectively. Sediment samples from NH, DH and ZH were distributed in the lower left, upper left and lower right parts, respectively. The PCA results showed significant differences in species composition among the groups and less differences within the groups.

**Figure 3:**
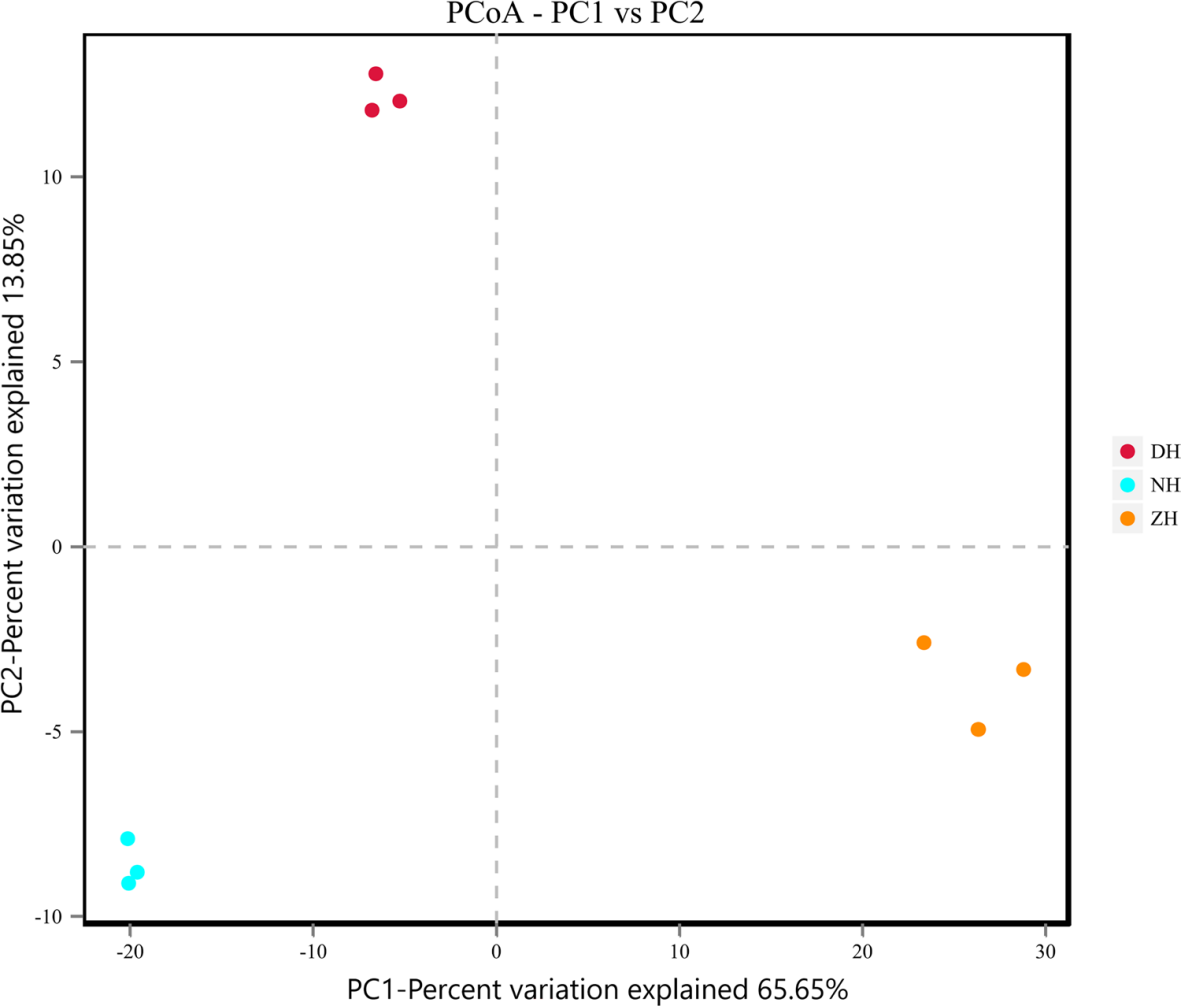
PCoA based on unweighted unifrac distance.

The Beta diversity of the samples was analyzed on QIIME to compare species diversity among samples. PCoA and UPGMA based on unweighted unifrac were conducted to identify whether species compositions were similar between samples. The PCoA images (Fig. 3) show the two axes can explain 65.65% and 13.85% of sorting results respectively. The PcoA results show that the species composition difference between groups is significant, and the difference within the group is small.

**Figure 4:**
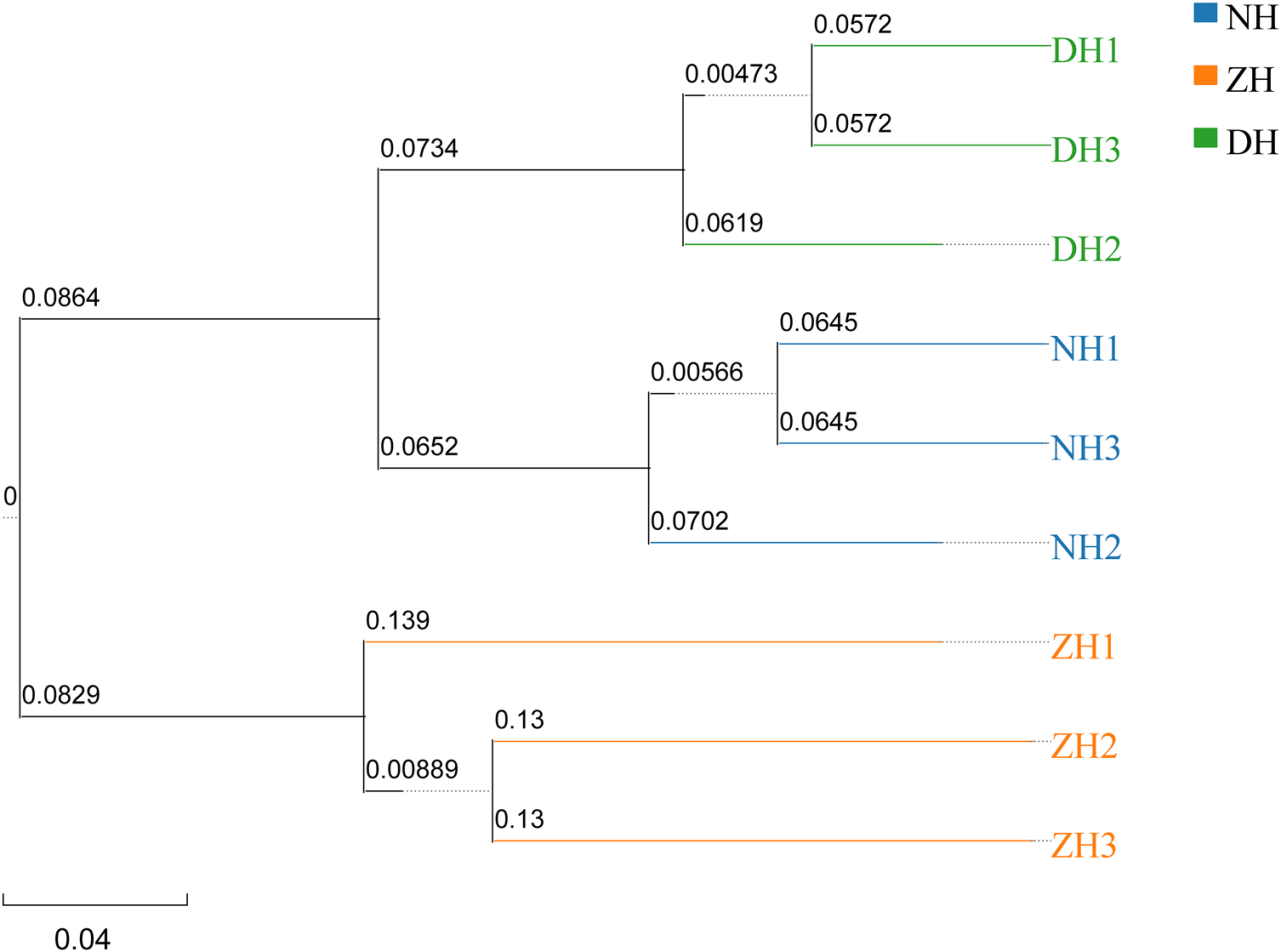
UPGMA cluster tree based on unweighted unifrac distance.

The hierarchical clustering of 9 samples is illustrated in Fig. 4, which is similar to the PCoA results. Clearly, the similarity of species compositions from samples in DH, NH and ZH is significantly different. DH1 and DH3, NH1 and NH3, and ZH2 and ZH3 all cluster. The samples from ZH are farther from the samples in the other two lakes on the cluster map, indicating samples from ZH are largely different from other sediment samples in terms of microbial communities.

### Bacterial communities of surface-sediments from Huaibei collapse lakes

**Figure 5:**
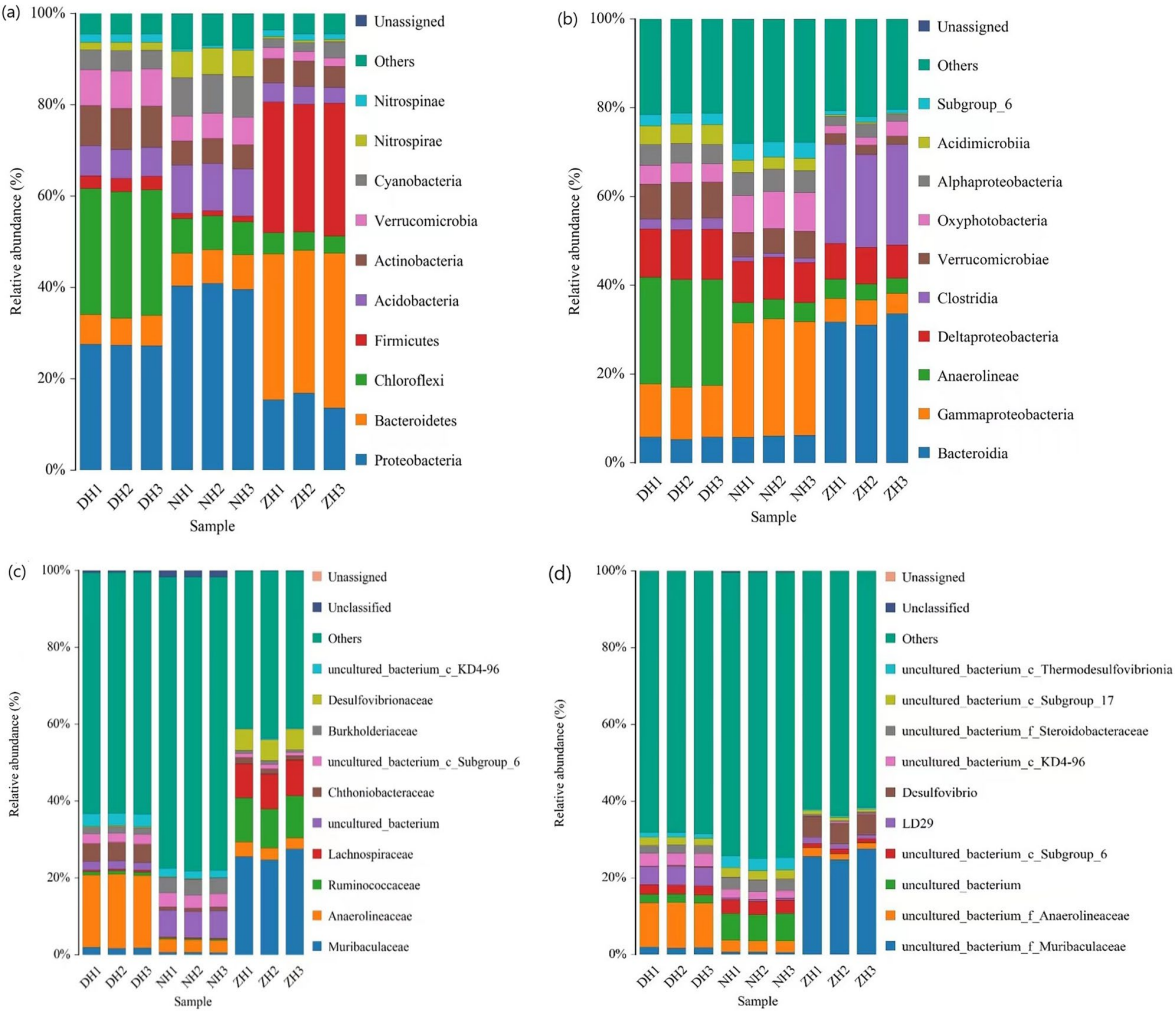
Relative abundance of dominant bacteria in surface sediments based on phylum, class, family, and genus levels.

The relative abundance at the level of bacterial phyla in surface sediments from different sampling sites is shown in Fig. 5a. The top 10 phyla ranked by relative abundance at the taxonomic phylum level are Pseudomonadota (13.64–40.90%), Bacteroidales (5.90–33.85%), Chloroflexales (3.80–27.75%), Firmicutes (1.14–29.07%), Acidobacteriales (2.71–10.55%), Actinobacteria (1.76–9.03%), Verrucomicrobiota (1.87–8.25%), Cyanobacteria (1.94–8.83%), Nitrospiria (0.40–5.80%), and Nitrospinae (0.49–1.84%). The dominant bacterial phyla are significantly different among the samples from different collapse lakes. The dominant bacterial phyla with relative abundance > 10% are Pseudomonadota (27.38% ± 0.14%) and Chloroflexales (27.59% ± 0.11%) in the sediments of DH; Pseudomonadota (40.28% ± 0.53%) and Acidobacteriales (10.39% ± 0.12%) from the sediments of NH; Pseudomonadota (15.30% ± 1.32%), Bacteroidales (32.36% ± 1.09%) and Firmicutes (28.57% ± 0.45%) in sediments from ZH.

The top 10 dominant phyla ranked by average relative abundan were sent to ANOVA to evaluate the differences in bacterial communities among the collapse lakes. Pseudomonadota is a dominant phylum in all sediment samples, with average relative abundance over 15%, but the relative abundance is very significantly different among the collapse lakes (*p* < 0.001), as it maximizes to 40.28% ± 0.53% in the sediments from NH. The average relative abundance levels of Actinobacteria (8.90% ± 0.08%), Chloroflexales (27.59% ± 0.11%), Nitrospinae (1.81% ± 0.02%) and Verrucomicrobiota (8.06% ± 0.18%) in the sediments from DH are all significantly higher compared with both NH and ZH (*p* < 0.001). The average relative abundance levels of Pseudomonadota (40.28% ± 0.53%), Acidobacteriales (10.39% ± 0.12%), Cyanobacteria (8.61% ± 0.15%) and Nitrospiria (5.78% ± 0.02%) in the sediments from NH are all significantly higher compared with both DH and ZH (*p* < 0.001). The average relative abundance levels of Bacteroidales (32.36% ± 1.09%) and Bacillota (28.57% ± 0.45%) in the sediments from ZH are both significantly higher compared with both DH and NH (*p* < 0.001). The differences in dominant phyla lead to significant differences in bacterial community compositions among the surface sediments from the three collapse lakes.

Figure 5b demonstrates the relative abundance of bacteria at the class level in the surface sediments from different sampling sites. The top ten classes ranked by relative abundance at the taxonomic class level are Bacteroidia (5.2–33.6%), Gammaproteobacteria (4.6–26.4%), Anaerolineae (3.4–24.2%), Deltaproteobacteria (7.5–11.3%), Clostridia (0.9–22.6%), Verrucomicrobiota (1.9–8.3%), Chroococcales (1.3–8.7%), Alphaproteobacteria (1.6–5.2%), Acidimicrobiia (0.2–4.4%) and Subgroup 6 (0.9–3.7%). Specific classes separate the three collapse lakes. The dominant bacterial classes with relative abundance > 10% in the sediments are Gammaproteobacteria (11.77% ± 0.20%), Anaerolineae (24.05% ± 0.20%), and Deltaproteobacteria (11.13% ± 0.20%) from DH; Gammaproteobacteria (25.94% ± 0.40%) from NH; Bacteroidia (32.12% ± 1.32%) and Clostridia (21.98% ± 0.90%) from ZH.

ANOVA shows the average relative abundance levels of Anaerolineae (24.05% ± 0.20%), Deltaproteobacteria (11.13% ± 0.20%), Verrucomicrobiota (8.06% ± 0.22%), and Acidimicrobiia (4.35% ± 0.13%) in the sediments from DH are all significantly higher compared with both NH and ZH (*p* < 0.001). The average relative abundance levels of Gammaproteobacteria (25.94% ± 0.40%), Chroococcales (8.46% ± 0.21%) and Subgroup_6 (3.59% ± 0.14%) in the sediments of NH are significantly higher compared with the other two lakes (*p* < 0.001). The average relative abundance levels of Bacteroidia (32.12% ± 1.32%) and Clostridia (21.98% ± 0.90%) in surface sediments of ZH are significantly higher compared with the other two lakes (*p* < 0.001).

The maps of top 10 family and genera in terms of relative abundance are shown in Fig. 5c,d respectively. Except for the unclassified bacteria, the bacterial community compositions are highly differentiated among the three lakes at levels of both family and genera. The dominant family of bacterial communities in the sediments from DH is Anaerolineaceae (18.99% ± 0.27%), followed by Chthoniobacteraceae (4.79% ± 0.10%) and uncultured_bacterium_c_KD4-96 (3.30% ± 0.06%), and the most abundant genus is uncultured_bacterium_f_Anaerolineaceae (11.68% ± 0.15%). The distributions of bacterial communities in the sediments from NH are significantly different from those of the other two lakes, but no species with relative abundance above 7%. The predominant species include uncultured_bacterium (6.97% ± 0.18%), Burkholderiaceae (4.10% ± 0.05%), uncultured_bacterium_c_Subgroup_6 (3.52% ± 0.14%), and Anaerolineaceae (3.30% ± 0.14%). The predominant microbial families in the sediments from ZH are Muribaculaceae (25.96% ± 1.48%), Ruminococcaceae (10.89% ± 0.66%), Lachnospiraceae (9.08% ± 0.26%) and Desulfovibrionaceae (5.49% ± 0.04%), and the most abundant genus is uncultured_bacterium_f_Muribaculaceae (25.96% ± 1.21%).

### Correlations between physicochemical factors of sediments and predominant communities

**Figure 6:**
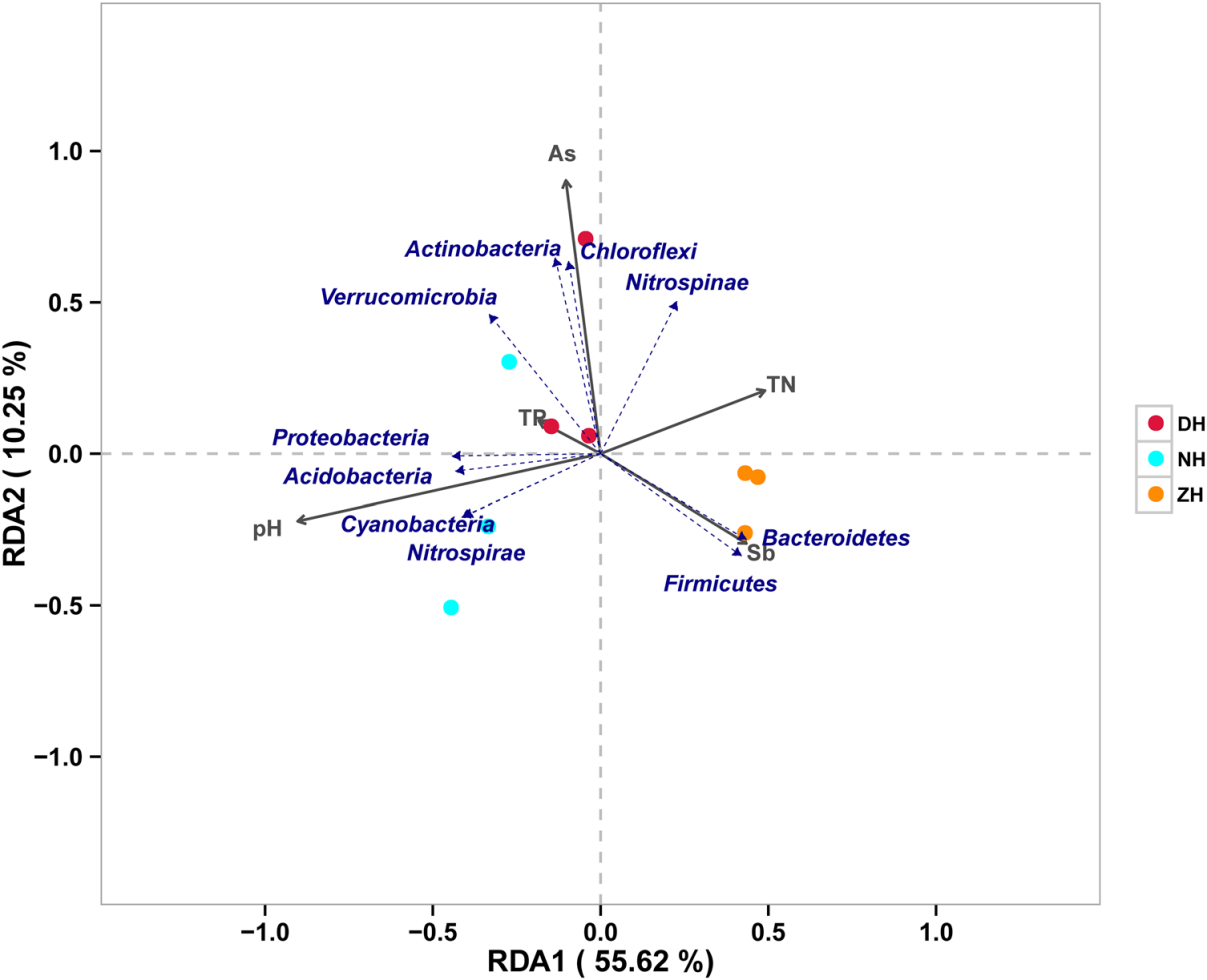
Redundancy analysis of horizontal dominant bacteria and physicochemical factors in sediments.

RDA was conducted to clarify the correlations between predominant phyla of bacteria and physicochemical factors of sediments (Fig. 6). The first and second principal axes account for 55.62% and 10.25% of information respectively. The environmental physicochemical factors positively correlated with the first principal axis include TN and Sb, and the negatively correlated factors are pH, TP, and As. The factors positively correlated with the second principal axis include As, TP, and TN, and the negatively correlated factors are pH, and Sb.

Pseudomonadota, Acidobacteriales, Cyanobacteria and Nitrospiria are all correlated positively with pH and TP, and significantly and negatively with TN. Firmicutes, and Bacteroidales are correlated significantly and positively correlated with Sb, and significantly and negatively with TP. Actinobacteria, and Chloroflexales are correlated significantly and positively with As, and negatively with Sb. Verrucomicrobiota is correlated more with TP, and negatively with Sb. Nitrospinae is affected jointly by As and TN, and is negatively correlated with pH.

## Discussion

The lake ecosystem is closely related to the microorganisms in the sediment. The nutrients in the lake affect the survival and reproduction of microorganisms, and the nutrients eventually settle on the bottom soil layer and are transformed. The assimilation and dissimilation of microorganisms in the sediment together with the action of microorganisms will bring nutrients from the sediment back into the water column^20^. Thus, interpreting and probing into the compositions of bacterial communities in sediments as well as the responses of sentinel dominant bacteria to environmental factors can characterize the operation modes of ecosystems and biogeochemical circulation. Herein, the compositions and species diversity of bacterial communities in sediments collected from three collapse lakes (DH, NH, ZH) of Huaibei were profoundly studied by high-throughput sequencing.

Microbiomes are a diverse and complex system, in which thousands of species of microbes live. These microbes evolve together with the host, and play critical roles in ecosystem restoration and environmental protection. Microbial diversity seems to be a key factor that maintains the resilience of microbiomes. Unfavorable environmental factors, such as high-concentration heavy metals, will significantly decrease microbial diversity, which in turn will lower the stability of other ecosystems. Improving the restoring ability of microbes before microbial diversity is disturbed, or recovering the balancing ability of microbes after microbial diversity is disturbed will bring significant benefits and contributes to ecosystem restoration in the environment. The high diversity and abundance of microorganisms can stabilize and restore environmental changes^21,22^. Fluctuations over time (seasonal patterns) and space (thermal layers) may affect the ratio between different ecotypes, but the OTUs in the community remain detectable throughout the year. According to the diversity indices, the ACE index and Shannon indices are both significantly different among different lakes (*p* < 0.05). Combining the results of PcoA and UPGMA hierarchical clustering analysis, we find the bacterial community structures among the three coal mining subsidence lakes have obvious geographical areas. The difference may be related to the formation of lakes. DH, ZH, and NH were formed by the collapse of Shuoli Mine, Zhahe Mine, and Yangzhuang Mine respectively. The physical and chemical properties of coal gangue in different mining areas are different, and the accumulation of a large amount of coal gangue at the lake bottom results in the nature of lake sediments. There are differences, which in turn affect the bacterial community structure and diversity in the sediments. Moreover, NH has been developed into a wetland park, which is more disturbed by human factors than the other two lakes. Comparison of Shannon index demonstrates that diversity of collapse lakes in Huaibei surpasses the sediment microbial communities in natural ponds of Cerrado Bay, Brazil, and the two major freshwater lakes of Yunnan province, China (Dian Lake and Erhai Lake)^21,23^. The reason for the abundant bacterial diversity in the sediments of the collapsed lakes of Huaibei may be similar to the rhizosphere effect, and the existence of large aquatic plants in the collapsed lake has a promoting effect^24^.

Our study shows that the average relative abundance levels of Pseudomonadota are 27.38% ± 0.14% and 40.28% ± 0.53% respectively. The average relative abundance of Pseudomonadota in the 9 sediment samples exceeds 15%, but the difference among the collapsed lakes is extremely significant (*p* < 0.001). Pseudomonadota are ubiquitous in sediments and play key roles in metabolism and sedimentation^25^. Other dominant groups in the sediments of the collapsed lakes in Huaibei also include Bacteroidales (5.90–33.85%), Chloroflexales (3.80–27.75%), Firmicutes (1.14–29.07%), and Acidobacteriales (2.71–10.55%). Similarly, another study demonstrates that Pseudomonadota is the richest phylum in all sediments, with average relative abundance over 30%, and other relatively abundant phyla in sediments include Nitrospiria, Acidobacteriales, Firmicutes, Bacteroidales, Chloroflexales, Cyanobacteria and Actinobacteria^26^. A study on bacterial community composition in surface sediments from East Lake of Wuhan shows that Pseudomonadota is relatively the most abundant, and other dominant phyla are Bacteroidales, Chloroflexales and Cyanobacteria^27^. These results are consistent with the bacterial spectrum in our study, but the compositional abundance is different. In this study, the bacterial groups observed in the sediments of the collapsed lakes of Huaibei are roughly the same as the bacterial lineages observed in the above studies. The relatively abundant phylum in the sediments of ZH is Bacteroidales (32.36% ± 1.09%), followed by Bacillota (28.57% ± 0.45%), which are different from the above studies, indicating that the bacteria in the sediments of the collapsed lakes in Huaibei are rich in diversity. In this study, Cyanobacteria was detected in all 9 sediment samples, and the relative abundance of Cyanobacteria (8.61% ± 0.15%) in the sediments of NH was significantly higher than that of the other two lakes (*p* < 0.001). As the NH is in the Nanhu Wetland Park, it is more severely disturbed by human factors than the other two lakes. As a result, the lake has been polluted and has become eutrophicated providing nutrients for the growth and reproduction of Cyanobacteria.

Moreover Gammaproteobacteria, Anaerolineae, and Deltaproteobacteria are the dominant bacteria in the sediments of DH; Gammaproteobacteria dominates the sediments of NH. Gammaproteobacteria usually exists in the sediments with high organic matter content. The high abundance of Gammaproteobacteria in the sediments of the NH shows that the organic matter content in the sediments is higher than that of the other two lakes. Moreover, Deltaproteobacteria is more widely distributed in freshwater sediments, which is because reduction by sulfates, plays key roles in sulfur circulation of sediments^28,29^. Anaerolineae belonging to Chloroflexales can biodegrade organic pollutants^30^, so the relatively higher abundance of Anaerolineae in sediments of DH reflects the higher concentrations of organic pollutants. Bacteroidia (32.12% ± 1.32%) and Clostridia (21.98% ± 0.90%) contribute to over 50% of bacteria in sediments from ZH. Reportedly, Bacteroidales is the second dominant group in the lake of the Third Pole Region(Mount Qomolangma region) belonging to Gram-negative bacteria, which is widely present in both terrestrial and aquatic environments and is an important part of the biodegradation of polymer organic matter^31,32^. Clostridia belonging to Bacillota generally inhabits terrestrial environments, but under abnormal conditions, can produce spores to provide the optimal adaptation strategy under various extreme environments^31^. The high abundance of Bacteroidia and Clostridia in the sediments of ZH is very important for maintaining its ecological function and nutrient cycle. The value of urban lakes lies more in flood storage, climate regulation, improvement of urban ecological environment and recreational tourism. Compared with natural lakes, urban lakes are more influenced by human activities, and human activities such as fish farming, shipping, sewage discharge, and fertilizer application critically impact the ecosystem health of lakes. At the same time, urban lakes need to assume more ecological services. Lake microorganisms are the subject of environmental remediation after lake pollution. Since collapse lakes are not natural, studying the microorganisms in the sediments therein is more practical.

As the habitat of bacteria, sediments have physical and chemical factors that will affect the community structure of bacteria to varying degrees, including TN, TP, pH^8,9^. Based on RDA (Fig. 6), the relationship between the phylum-level dominant bacterial communities and the physiochemical factors of sedimentation was explained in the collapsed lake area of Huaibei. As reported, environmental factors including nitrates and phosphates considerably impact the bacterial community compositions of sediments in Laurentian Great Lakes^33^. TN concentration remarkably affects bacterial communities in sediments, and significant correlations are found between Nitrospiria and TN concentration, between Pseudomonadota and TP (positive), and between Acidobacteriales and pH^26^. Waters with different pHs were selected for different bacterial assemblages^34^. Our research results are consistent with the conclusions of the above-mentioned researchers. The pH of sediments may alter the H^+^ and OH^-^ balance on cell membranes or walls of bacteria, impacting the propagation and metabolism of microbes, so the bacterial community compositions may differ among lake sediments at different pH^35^. Sediments are the main living environment for bacterial communities, and significant differences in pH can affect the bacterial communities living in them. pH can cause changes in cell membrane charge, which affects the absorption of nutrients by microorganisms and the activity of enzymes in the metabolic process; changing the availability of nutrients and the toxicity of harmful substances. The pH of the medium not only affects the growth of microorganisms, but even the morphology of microorganisms. Microbial metabolic activities also change the environmental pH, and the metabolism of microorganisms also has a certain effect on the pH of sediments. Our study shows that pH is significantly different between the sediments of ZH and NH (*p* < 0.05), so the average relative abundance levels of Pseudomonadota (40.28% ± 0.53%), Acidobacteriales (10.39% ± 0.12%), Cyanobacteria (8.61% ± 0.15%), and Nitrospiria (5.78% ± 0.02%) in sediments from NH are all significantly higher compared with both DH and ZH (*p* < 0.001), which are consistent with the RDA results. Sb and As concentrations in sediments were also measured. RDA demonstrates significant positive correlations between Actinobacteria or Chloroflexales and As, and between Firmicutes or Bacteroidales and Sb. Nitrospinae are jointly affected by As and TN. Studies prove that heavy metals are among the key influence factors on bacterial community compositions^36,37^. A study on the effects of heavy metals at a 12-concentration gradient (Cd, Pb, Cu, Ni) on microbial community composition in south of Poland shows that Actinobacteria, Chloroflexales, Bacteroidales and Planctomycetes are very sensitive to changes in heavy metals^38^. It can be guessed that Actinobacteria and Chloroflexales are easier to adapt to environments with higher As content, while Firmicutes and Bacteroidales are easier to adapt to environments with higher Sb content.

The microbial community composition is affected by many factors. Macroscopically, these factors are divided into human activities, and non-human activities. Human activities affect the compositions of microbial communities in lake sediments. The microbial communities in the disturbed zone of human activities are more diverse than those in the un-disturbed zone. Specially, the microbial communities in South Lake are more diverse than those in East Lake and Middle Lake, but the main bacterial phyla of the communities are similar among lakes. The non-human activities are mostly environmental factors, such as geographical locations, space–time, and space position (depth of sediments in the lake)^39^. The relationships between environmental factors and sediment bacteria should be further investigated.

## Conclusions

The physicochemical properties of sediments sampled from three collapse lakes in Huaibei were measured, and the diversity sequencing and species compositions of bacteria were analyzed. Results showed Anaerolineae was the most abundant class in the sediments of East Lake, Gammaproteobacteria was dominant in the sediments of South Lake, and Bacteroidia and Clostridia contributed to over 50% of bacteria in the sediments of Middle Lake. The community diversity of sediments in East Lake was higher compared with the other two lakes, and the bacterial species compositions of sediments significantly differed among the lakes. The influence factors on the species compositions were also analyzed. The main environmental factors that affected the bacterial community composition of sediments in collapse lakes included pH, TN and TP, followed by Sb and As. These conclusions were also validated by RDA. The bacterial community compositions among the collapse lakes were compared, which is a scientific basis for future research on the physiology and ecology of collapse lakes in coal mines and on subsequent ecological restoration in collapse lakes.

## Materials and methods

### Study area description and sample collection

Huaibei, located in the north of Anhui Province, is one of the bases of coal supply in China. However, coal mining has resulted in multiple collapses, affecting morphological characteristics and forming several collapse zones. Long-term impoundment in the collapse zones has formed several lakes, mainly including East Lake, South Lake, and Middle Lake. The bottom sediments of collapse lakes are compositionally complex, mainly including coal gangue and flyash, and have formed unique aquatic ecosystems.

Table 3 shows the sampling points and geographical locations of the Huaibei coal mining subsidence lake area. On May 8, 2019, surface sediments (0–20 cm depth) were collected from DH, NH, and ZH. Three parallel sites were set up in each lake, with a total of 9 sampling points. Among them, the water area of DH is about 1.91 km^2^, formed by the collapse of the Suli Mine. The sampling points are DH1, DH2, and DH3. The water area of ZH is 7.77 km^2^, with a total storage capacity of 36.8 million m^3^, formed by the collapse of the Zhahe Coalfield. The sampling points are ZH1, ZH2, and ZH3. Located in the Nanhu Wetland Park, NH is more affected by human factors than the other two lakes sampled. It was formed by the collapse of the Yangzhuang Coal Mine. The water area is 3.35 km^2^. NH1, NH2 and NH3 are set as sampling points. Surface sediments from each sampling site were collected by using a grab-bucket dredge, with three times of sampling at each site. The sample from each sampling time was divided into two parts, which were put into centrifuge tubes. The tubes were sealed by aseptic sealing bags, frozen, immediately sent to our laboratory, and cold-stored. The whole procedures were conducted under aseptic conditions. One part of the samples were naturally air dried in the laboratory for measurement of physicochemical indicators. The other part of samples were frozen in dry ice and sent to Beijing Biomarker Technologies Co., Ltd. for analysis of bacterial diversity.

**Table 3:** Sampling sites.

Sampling site	Codes	Latitude	Longitude	Description
East Lake	DH 1	N 33° 57′ 40″	E 116° 51′ 40″	Water area 2850 Mu, resulting from collapse of Shuoli coal mine
	DH 2	N 33° 57′ 59″	E 116° 52′ 16″	
	DH 3	N 33° 57′ 59″	E 116° 52′ 15″	
Middle Lake	ZH 1	N 33° 54′ 56″	E 116° 48′ 33″	Zhahe coalfield collapse zone with water area of 11,600 mu, total impoundment volume of 36.8 million m^3^
	ZH 2	N 33° 55′ 1″	E 116° 48′ 31″	
	ZH 3	N 33° 55′ 1″	E 116° 48′ 30″	
South Lake	NH 1	N 33° 54′ 5″	E 116° 48′ 38″	Resulting from of Yangzhuang coal mine collapse, with water surface area of 5000 mu
	NH 2	N 33° 54′ 6″	E 116° 48′ 47″	
	NH 3	N 33° 53′ 58″	E 116° 48′ 58″	

### Measurement of physicochemical factors of sediments

Physicochemical factors of the 9 sediment samples were analyzed. The pH of sediments was detected according to an international standard ISO10390:2005 soil quality pH detection^40^. TN was measured by Kjeldahl's method^41^ and TP was detected by alkali-melted Mo Sb colorimetric method^42^. As and Sb concentrations were measured by an AFS-8220 atomic fluorescence spectrophotometer (Ji Tian Instrument Co., Beijing, China).

### DNA extraction and high-throughput sequencing of sediments

From each of the 9 sediment samples, DNA was extracted three times using MN NucleoSpin 96 Soil kits as per the manufacturer's introductions, and DNA quality was detected by agarose gel electrophoresis. The V3-V4 high-variable zones of 16S rRNA genes were amplified by polymerase chain reaction (PCR) using forward primer 338F (5′-ACTCCTACGGGAGGCAGCA-3′) and reverse primer 806R (5′-GGACTACHVGGGTWTCTAAT-3′)^43^. The PCR amplification system was 10 μL, including 50 ng ± 20% genomic DNA, 0.3 μL of each of forward and reverse primers (10 μM), KOD FX Neo Buffer 5 μL, dNTP (2 mM each) 2 μL, and KOD FX Neo 0.2 μL, which were diluted by ddH2O to 10 μL. The PCR conditions were: pre-denaturation at 95 °C × 5 min; denaturation at 95 °C × 30 s, annealing at 50 °C × 30 s, extension at 72 °C × 40 s, 20 cycles; extension at 72 °C × 7 min and retension at 4 °C. After that, PCR purification products in the target zones were sent to Solexa PCR. The reaction system was 20 μL, which included PCR purification products from the target zone (5 μL), ^★^MPPI-a(2 μM) and ^★^MPPI-b(2 μM) (each 2.5 μL), and 2 × Q5 HF MM 10 μL. The reaction conditions were: pre-denaturation at 98 °C × 30 s; denaturation at 98 °C × 10 s, annealing at 65 °C × 30 s, extension at 72 °C × 30 s, 10 cycles; extension at 72 °C × 5 min. PCR products were mixed at mass ratio of 1: 1 according to the quantitative results of electrophoresis, and purified on columns using an OMEGA DNA purification kit. After 1.8% agarose gel electrophoresis at 120 V for 40 min, the target fragments were recovered using a Monarch DNA gel extraction kit and sent to Beijing Biomarker Technologies Co., Ltd. for base construction and sequencing.

On an Illumina HiSeq sequencing platform, a small-fragment library was built by paired-end sequencing, and microbiological diversity in the sediment samples was sequenced. After quality check, the library was sent to double-end sequencing on Illumina HiSeq 2500. The original images acquired from the sequencing platform were converted by base identification and analysis into original sequences.

### Sequencing data processing

With FLASH 1.2.11^44^, the reads of the original data were spliced at the minimum overlap 10 bp and overlap zone maximum mis-matching ratio of 0.2 (default), forming original Tags data (Raw Tags). On Trimmomatic 0.33^45^, the FASTQ double-end sequences in the base quality format of phred33 or phred64 were sent to quality filtration at 50 bp window. If the within-window average quality was smaller than 20, the back-end bases starting from the window were cut off. The Tags after quality control and in length less than 75% Tags length were filtered out, forming Clean Tags. Chimeras were removed on UCHIME 8.1^46^, forming the final valid data.

At the similarity level of 97%, the Tags were clustered using UCLUST^47^ on QIIME 1.8.0^48^, and operational taxonomic units (OTU) were filtered at the threshold of 0.005% of all tested sequences^49^. On RDP Classifier at the confidence level of 0.8^50^ the OTUs were taxonomically annotated in accordance with Silva^51^ taxonomic database (Release128, http://www.arb-silva.de). Species abundance maps at different taxonomic levels were plotted on QIIME, and community composition maps were drawn on R-language.

As for amplicon sequence, two methods viz. ASV and OUT are commonly used to decrease the effect of sequencing errors. OUT is to compute the similarity of each pair of sequences, and then clusters at the threshold of 97% similarity, forming different taxons. ASV first discards the mistaken sequences and then clusters by setting the standard of Identity at 100%.

ASV has some limitations. Firstly, some species at very low abundance in the samples may be treated as wrong sequences and thus discarded. Secondly, it uses a smaller number of valid Reads than OTU does. Lastly, the premise for ASV is that the data shall be sufficient for the construction of an appropriate error model that can accurately detect the wrong sequences. OTU can rapidly form an abundance matrix, it is independentn the reference database, especially when the samples contain very few known species. OUT is to compute the similarity of each pair of sequences, and then clusters at the threshold of 97% similarity, forming different taxons. Therefore, OTU is mainly chosen as amplicon sequence.

### Statistical analysis

The between-sample superposition of OTUs was visually displayed as Venn Diagram^52^ to find out the common microbes among different environments. The diversity indexs include Shannon index, and Simpson index each sample was evaluated on Mothur 1.30^53^, and coverage rates of OTUs were statistically analyzed to ensure sequencing depth. The differences in α diversity index among collapse lakes were assessed by T-test. The β diversity index was calculated on QIIME, and similarity levels in species diversity among samples were compared. Results were analyzed on R-language by principal co-ordinates analysis (PCoA) and unweighted pair-group method with arithmetic means (UPGMA), which are based on unweighted unifrac. Significant levels about differences in abundance of microbial communities among samples were tested by analysis of variance (ANOVA) at different taxonomic levels. Associations between physicochemical factors of sediments and microbial communities were assessed using redundancy analysis (RDA) conducted on a BMKCloud biological cloud computing platform and Pearson correlation analysis.

## Data Availability

The rawdata sequencing has been uploaded to the NCBI database, and the gene sequence accession number is SRP329367.
